# Two Military Hospital Gazettes

**Published:** 1916-09-23

**Authors:** 


### Two Military Hospital Gazettes.

### The "Southern Cross" in September.

There is considerable variety in the current issue of the *Southern Cross*, now well known as the monthly journal of the 1st Southern General Hospital, Birmingham. The cartoons in the "Who's Zoo" series continue to appear, and Pepys is still behind the scenes, to the amusement of his readers. The public memory is usually so short that it is rather curious to come across a poem entitled "Turning the Other Cheek," in which "a reverend pedagogue" is chaffed for having suggested the "neutralisation of Gibraltar." There are numerous illustrations, consisting of drawings and photographs, and the new series of "Muddley Road Celebrities" starts well with the black-and-white drawing of "Jacko," in the regulation costume of a cook. Some news is given of "old friends," and, as they are necessarily scattered and variously employed, it is to be hoped that they will remember their old hospital and continue to send accounts of their doings. Such articles can be among the most interesting sent in, and are always read with eagerness.

### An Ambitious Number.

The September number of the *Craigleith Hospital Chronicle* maintains the high standard which we have often admired. In paper, printing, and variety of contents it is one of the most ambitious of hospital gazettes, a pleasure alike to read and handle. On the principle that whatever is interesting is likely to interest its readers, it does not hesitate to leave the beaten track. Thus Miss Mary R. Walker contributes an article on "The Free Kindergarten and the War," in which the warlike games of the children are described and delightfully illustrated by the art mistress and some of the pupils of St. George's High School. Comic verses, articles, and sketches are accompanied by an article on "Sphagnum Moss: Its Use in Surgery," an officer's letter from the Western front to his children, and a story of the battle of Waterloo, which was "written in 1883" by H. W. Le Mesurier; while Dr. J. A. MacDougall continues his series on the Scottish regiments by an article on "The Royal Scots Fusiliers." What more could anybody want?

